# Sleep disorders in patients with juvenile idiopathic arthritis as assessed by the sleep disturbance scale for children

**DOI:** 10.1186/1546-0096-9-S1-P148

**Published:** 2011-09-14

**Authors:** RP Khubchandani, AA Bagde, A Pistorio, RP Hasija

**Affiliations:** 1 Pediatric Rheumatology Clinic, Jaslok Hospital, Mumbai, India; 2 IRCCS G Gaslini, Servizio di Epidemiologia e Biostatistica, Genova, Italy

## Background

Sleep disturbances in children with Juvenile Idiopathic Arthritis (JIA) are an understudied facet of the disease.

## Aim

We therefore studied the incidence and types of sleep disorders in them using the Sleep Disorder Scale for Children (SDSC)

## Methods

Out of 35 patients with JIA, enrolled after parental consent, 31 (23 males and 8 females) completed the study. Mean age of the patients was 9.8 years (Range 6.1-14.5 years). The patients were enrolled when they demonstrated active disease at presentation or during relapse, as 'active disease group' and followed up regularly at 6-8 weekly intervals, till they improved with treatment. Disease activity of JIA was assessed by ACR PEDI criteria. Sleep was studied using SDSC which is a validated screening tool (for the age interval studied), that categorizes sleep disturbances into six different categories: Disorders of initiating and maintaining sleep (DIMS), Sleep disordered breathing (SDB), Disorders of arousal (DA), Sleep wake transition disorders (SWTD), Disorders of excessive somnolence (DOES) & Sleep hyperhydrosis (SHY). The SDSC questionnaire was filled by the parent / patient in the waiting room at each follow up visit. When the patients showed maximum improvement in JIA, they were included in the 'post treatment' group. For quantifying sleep disorders, we compared the scores of the subjects with scores derived from a sample of normal population in a similar age band from our city. Differences between the active and post treatment groups were analyzed by non parametric Wilcoxson test for paired data and non parametric Mann-Whitney U test for unpaired data

## Results

The difference between *total* SDSC scores of 'active' group and 'post treatment' group was statistically significant (p<0.05). Significant differences were also noted in all the 6 *individual* categories of sleep disorders. Age and gender did not influence SDSC scores.

Based on the data derived from the SDSC scores obtained from normal children in the same age group from our population, 12/31 (39%) patients demonstrated an abnormal total SDSC score in the active group ( > mean + 2SD). In the post-treatment group no patient had abnormal total SDSC score.

Individual sleep disorders were more common with frequencies of: DOES 25, SHY 14, DIMS 6, DA 6 and SBD 1. 10 reported 1 sleep disorder and 19 reported more than 1 disorder.

Out of 31 patients, 19, 6, 4 & 2 improved by ACR PEDI 90, 70, 50 and 30 respectively. The mean improvement in total SDSC scores in these ACR groups was seemed to correlate with the improvement in ACR criteria with patients who improved by ACR 90 showing maximum change in SDSC scores and those in the ACR 30 showing a minimum change. The number of patients in some of these ACR groups was too small to derive statistical significance.

## Conclusion

Multidimensional sleep disorders are common in JIA. Sleep disorders of all categories as classified by the SDSC reverse as the JIA improves. The improvement in sleep disturbances bears a correlation with the improvement in JIA activity.

